# Neighbourhood food typologies, fast food outlet visitation and snack food purchasing among adolescents in Melbourne, Australia

**DOI:** 10.1017/S1368980021004298

**Published:** 2022-03

**Authors:** Venurs HY Loh, Maartje Poelman, Jenny Veitch, Sarah A McNaughton, Rebecca Leech, Anna Timperio

**Affiliations:** 1 Institute for Physical Activity and Nutrition, School of Exercise and Nutrition Sciences, Deakin University, Geelong Waurn Ponds Campus, 75 Pidgons Road, Waurn Ponds, Geelong, VIC 3216, Australia; 2 Chair Group Consumption and Healthy Lifestyles, Wageningen University & Research, Wageningen, The Netherlands

**Keywords:** Neighbourhood, Food environment, Fast food, Food purchasing, Adolescents

## Abstract

**Objective:**

Despite the increased attention on neighbourhood food environments and dietary behaviours, studies focusing on adolescents are limited. This study aims to characterise typologies of food environments surrounding adolescents and their associations with fast food outlet visitation and snack food purchasing to/from school.

**Design:**

The number of food outlets (supermarket; green grocers; butcher/seafood/deli; bakeries; convenience stores; fast food/takeaways; café and restaurants) within a 1 km buffer from home was determined using a Geographic Information System. Adolescents’ self-reported frequency of fast food outlet visitation and snack food purchasing to/from school. Latent Profile Analysis was conducted to identify typologies of the food environment. Cross-sectional multilevel logistic regression analyses were conducted to examine the relationships between food typologies, fast food outlet visitations and snack food purchasing to/from school.

**Setting:**

Melbourne, Australia.

**Participants:**

Totally, 410 adolescents (mean age= 15·5 (sd = 1·5) years).

**Results:**

Four distinct typologies of food outlets were identified: (1) limited variety/low number; (2) some variety/low number; (3) high variety/medium number and (4) high variety/high number. Adolescents living in Typologies 1 and 2 had three times higher odds of visiting fast food outlets ≥1 per week (Typology 1: OR = 3·71, 95 % CI 1·23, 11·19; Typology 2: OR = 3·65, 95 % CI 1·21, 10·99) than those living in Typology 4. No evidence of association was found between typologies of the food environments and snack food purchasing behaviour to/from school among adolescents.

**Conclusion:**

Local government could emphasise an overall balance of food outlets when designing neighbourhoods to reduce propensity for fast food outlet visitation among adolescents.

Overweight and obesity during childhood and adolescence is a global public health concern^(1)^. It has been established that individuals who are overweight in the early years are linked to obesity, chronic diseases and premature death in later years^(2)^. Although obesity and chronic diseases are largely preventable by healthy lifestyles, including a healthy diet, young people worldwide are increasingly consuming fewer healthy foods (e.g. fruits and vegetables, wholegrains and dairy) and more unhealthy foods (e.g. ultra-processed and energy-dense food)^(3–6)^. Fast food and pre-packaged snacks are major sources of energy-dense food intake, and consumption of these foods has increased substantially among adolescents in high-^(4,7)^ and low-to-middle-income countries^(8)^ in the past decades.

There are multiple socio-ecological factors that shape dietary behaviour among young people, including the local food environment^(9)^. Local food outlets facilitate the opportunity for residents to visit, purchase and consume both healthy and unhealthy food items^(9)^. Over the past decade, the food environment in Australia has changed remarkably, and there is an overabundance of unhealthy food options compared with healthy food options available^(10–12)^. For example, a recent study in Metropolitan Melbourne observed a shift towards a greater dominance of unhealthy food outlets relative to healthy food outlets across Melbourne from 2008 to 2016^(13)^. An abundance of energy-dense and unhealthy food has been shown to influence an individual’s propensity to choose healthy food options^(14)^.

The neighbourhood food environment may have a particularly strong influence on adolescents’ dietary behaviours as they may be more restricted than adults in terms of their ability to travel independently beyond their neighbourhood^(15)^. Adolescence is also a developmental period characterised by asserting their independence in relation to food choices and the need for social affirmation from peers^(16)^. However, studies that have explored neighbourhood food outlet exposure and dietary behaviours among adolescents are few and the findings are inconsistent. Studies among adolescents from the USA^(17,18)^ and Canada^(15,19)^ have found positive associations between the proximity, availability or density of unhealthy food outlets near home (fast food, convenience stores and corner stores) and the purchase of snack food and fast food, while other studies from the USA^(20)^ and Denmark^(21)^ found no associations between the proximity and availability of stores in neighbourhood food environment and the purchase of fast food and junk food.

A possible explanation for the mixed findings may be due to the heterogeneity of food environment exposure measures used across studies^(22)^. Geographic Information Systems are one of the most commonly used methods to assess food environments objectively. Geographic Information System-based measures have been used to assess availability of food outlets using binary (i.e. presence/absence) or non-binary (i.e. density, count) data as proxies for exposure. However, these measures may show different associations with dietary behaviours^(23)^. A common limitation of the literature is the assessment of only one or a limited selection of food outlets, usually fast food outlets or supermarkets, to assess the relationship between neighbourhood food outlet exposure and dietary intake or behaviours. However, other types of food outlets (e.g. cafes and bakeries) and the mix of food outlets available may influence food choice and shape dietary behaviour. Studies that have incorporated various food outlets using measures of relative availability (e.g. ratio or proportion of supermarkets to fast food outlets) have observed positive and more robust associations with dietary or weight-related outcomes than absolute availability of food outlets^(10,24,25)^. While this suggests that relative measures may be better predictors for dietary behaviour as they reflect the balance of the food environment, the use of relative measures has limitations^(26,27)^. For example, three convenience stores and a café would be treated the same as six convenience stores and two cafés; however, these food environments could have different associations with dietary behaviours.

Previous studies have found evidence to suggest that characteristics of the food environment tend to aggregate to form typologies^(27,28)^. Exploring clustering of different types of food outlets to develop typologies of food environments may offer a data-driven way to incorporate data on a range of outlets to determine influences on dietary behaviour. However, few studies have used data-driven approaches to characterise typologies within food environments^(29)^ and the relationship between these typologies and food choice and purchasing. One study conducted among children in Melbourne found that those exposed to environments close to home characterised as having a variety of food outlet types had a healthier dietary pattern during adolescence than those residing in a neighbourhood characterised as having few types of outlets (mainly convenience stores and cafes/restaurants)^(30)^. While this suggests that exposure to a range of food outlets may alter the impact of convenience stores and fast food outlets by providing more options, that study was based on presence or absence of food outlet types and did not consider the number of outlets.

To better reflect how food outlets cluster to form typologies, it is important to incorporate both the variety and quantity of food outlets near home. This study aimed to examine associations between typologies of neighbourhood food environments and fast food outlet visitation and snack food purchasing behaviour among adolescents living in metropolitan Melbourne, Australia.

## Methods

This study uses data from the Neighborhood Activity in Youth (NEArbY) study conducted between August 2014 and December 2015 among adolescents residing in Melbourne, Australia. The NEArbY study is part of the multicounty International Physical Activity and the Environment Network Adolescent project^(31)^. This study adhered to the STROBE-nut reporting guidelines (Appendix 1).

### School recruitment

School and participant recruitment have been detailed elsewhere^(32)^. Briefly, the selection of schools was based on statistical area level 1 (SA1) walkability and income quadrants in order to maximise heterogeneity in built environment and socio-economic position. Eighteen of 137 invited secondary schools consented to participate (18 % response rate). Participating schools selected year levels between years 7 and 12 to take part, and students were given a recruitment package, which consists of the study information, a parent survey and a consent form. A total of 528 students provided parental consent and student assent. Of these, 468 students completed an online survey at school and 473 had their residential addresses geocoded at the SA1 level. In total, 465 students had survey and residential address data. Parents also completed a survey, but it was only used here to supplement missing data for age and sex.

## Measures

### Fast food outlet visitation

Students reported how often they visit fast food outlets in a usual month using items adapted from Thornton *et al.* ^(33)^. The fast food outlets were McDonalds, KFC, Subway, Hungry Jacks, Red Rooster, Nando’s and ‘Others’. The response categories for visiting each fast food outlet, with scoring in parentheses, were ‘never/rarely’ (0), ‘once/fortnight’ (0·5), ‘1–2 times/week’ (1·5), ‘3–4 times/week’ (3·5), ‘5–6 times per week’ (5·5) and ‘at least once a day’ (7). Summary scores were computed by adding scores for each type of outlet. Due to the zero-inflated and left-skewed distribution, fast food visitation was then categorised into (i) once a week or more and (ii) less than once a week.

### Snack foods purchasing behaviours to and from school

Two items about purchasing ‘snack foods’ (defined as food eaten between meals, such as muesli bars, chocolates, pastries and lollies) were included. Participants were asked to report how often they bought snack foods to eat: (1) on the way to school and (2) on the way home from school. The response categories were ‘not in the last month’, ‘1–2 times/month’, ‘1–2 times/week’, ‘most days’ and ‘every day’. Due to the zero-inflated and left-skewed distribution, responses were dichotomised into (i) once a week or more and (ii) less than once a week for snack purchasing to school and from school separately.

### Neighbourhood food environment

Using ESRI ArcGIS 10·3 (Redlands, CA, US), the number of food retailers within a 1 km street network buffer around participants’ residential addresses, a distance deemed to be walkable according to adolescents^(34)^, was determined. Seven types of food retailers were examined: (i) supermarkets (supermarkets and ethnic grocers); (ii) green grocers; (iii) butchers, poultry and seafood; (iv) bakeries; (v) convenience stores; (vi) fast food outlets and major takeaways and (vii) café and restaurants. The locations of major supermarket chains and fast food outlets were obtained from company websites, butchers, poultry and seafood from PrimeSafe, and fast food outlets from company websites. All other categories of food retailers were sourced from Local Government food registries or phone directories (Yellow Pages, White Pages), where Local Government records were unable to be obtained.

### Socio-demographic variables

Adolescents self-reported their age and sex. The parent survey supplemented missing information on age (*n* 7). Neighbourhood disadvantage was determined at the SA1 level based on residential address using the Index of Relative Socioeconomic Disadvantage^(35)^. The IRSD score reflects each SA1’s overall level of disadvantage based on seventeen aspects that capture a range of socio-economic factors, including occupation, education, income, unemployment rate and household structure (among others). A higher IRSD score reflects a relatively advantaged area than an area with a lower score. The IRSD score in this sample ranged from 380 to 1137, with a mean of 995 (sd = 101·4).

### Statistical analyses

Of the 465 participants, those with missing data for age (*n* 9) and food purchasing behaviours to and from school (*n* 45), and whose 1 km network buffer was not covered by the food outlet mapping described earlier (*n* 1), were excluded. This reduced the analytic sample to 410 participants.

To identify neighbourhood typologies based on the seven types of food outlets within 1 km from home, latent profile analysis (LPA) with a Poisson link function was conducted using maximum likelihood estimation. LPA is a data-driven method of identifying groups of individuals (sub-populations) based on similarities in patterns within a set of variables^(36,37)^ and is capable of handling count data^(36)^. The method assigns individuals into a user-specified number of groups (latent classes) based on probability of group membership. The LPA input variables were the frequency counts of the seven food outlets within the 1 km buffer from participants’ home addresses. Then, the LPA models assigned participants to groups based on the number of food outlets. While each food outlet added to the LPA was a count, the typology derived is distinguishable by variety (the mix of different types of food outlets) and number of food outlets (count of food outlets). To determine the appropriate number of latent classes, a sequence of models with increasing numbers of classes (2–6 classes) were tested. These models were compared, and a combination of criteria was considered to select the most appropriate number of classes to represent typologies for this sample. These included model fit indicators generated by the LPA (i.e. the Akaike Information Criteria, Bayesian Information Criterion) and likelihood ratio statistical test methods, where lower values indicate better model fit, as well as practical criteria regarding the size of each class and the interpretability of the classes^(36)^

The ANOVA was conducted to assess whether neighbourhood disadvantage scores differ by neighbourhood food typologies. Separate multilevel logistic regression models were conducted to assess associations between neighbourhood food typologies, fast food visitations and snack purchasing behaviours. School (the unit of recruitment) was entered as a random effect variable to account for clustering. All models were adjusted for age, sex and neighbourhood disadvantage. The precise threshold to indicate statistical significance was not used in this study^(38,39)^. As such, 95 % CI and exact *P*-values are presented to indicate the level of evidence they provide: *P* < 0·005 providing strong evidence, *P* < 0·05 providing some evidence, .05 < *P* < 0·1 providing weak evidence and *P* ≥ 0·1 providing no evidence^(40)^. All analyses were undertaken using STATA/SE 15·0.

## Results

### Participant characteristics

Mean age was 15·5 (sd = 1·5) years and 244 (59 %) participants were girls. Overall, 47 % of adolescents visited fast food outlets at least once a week or more, 11 % bought snack foods on the way to school at least once a week or more and 21 % bought snack foods to eat on the way from school at least once a week or more.

### Neighbourhood food typologies

The six and five class solutions had the best fit based on the Akaike Information Criteria, Bayesian Information Criterion and the log likelihood values (Table 1). However, the cell sizes for some of the subgroups in these solutions were small (e.g. < 2 % of sample) and had similar characteristics between subgroups, making it difficult to interpret meaningful differences between them. Based on a combination of model fit and interpretability of the solution, the four-class solution was chosen as it had meaningful distinction between subgroups with reasonable cell sizes^(36)^.

**Table 1:** Comparisons of latent profile solutions of 2–6 according to the model fit indicators

Number of classes	2	3	4	5	6
Log likelihood	−5327·8	−4429·2	−4151·3	−3928·7	−3870·8
Degree of freedom	15	23	31	39	47
AIC	10 685·7	8904·5	8364·6	7935·5	7835·6
BIC	10 745·9	8996·8	8489·1	8092·1	8024·4
Cell sizes per subgroup	350/60	246/126/38	213/133/39/25	202/126/42/27/7	117/151/82/31/22/7

AIC, Akaike Information Criteria; BIC, Bayesian Information Criterion.

**Fig. 1:**
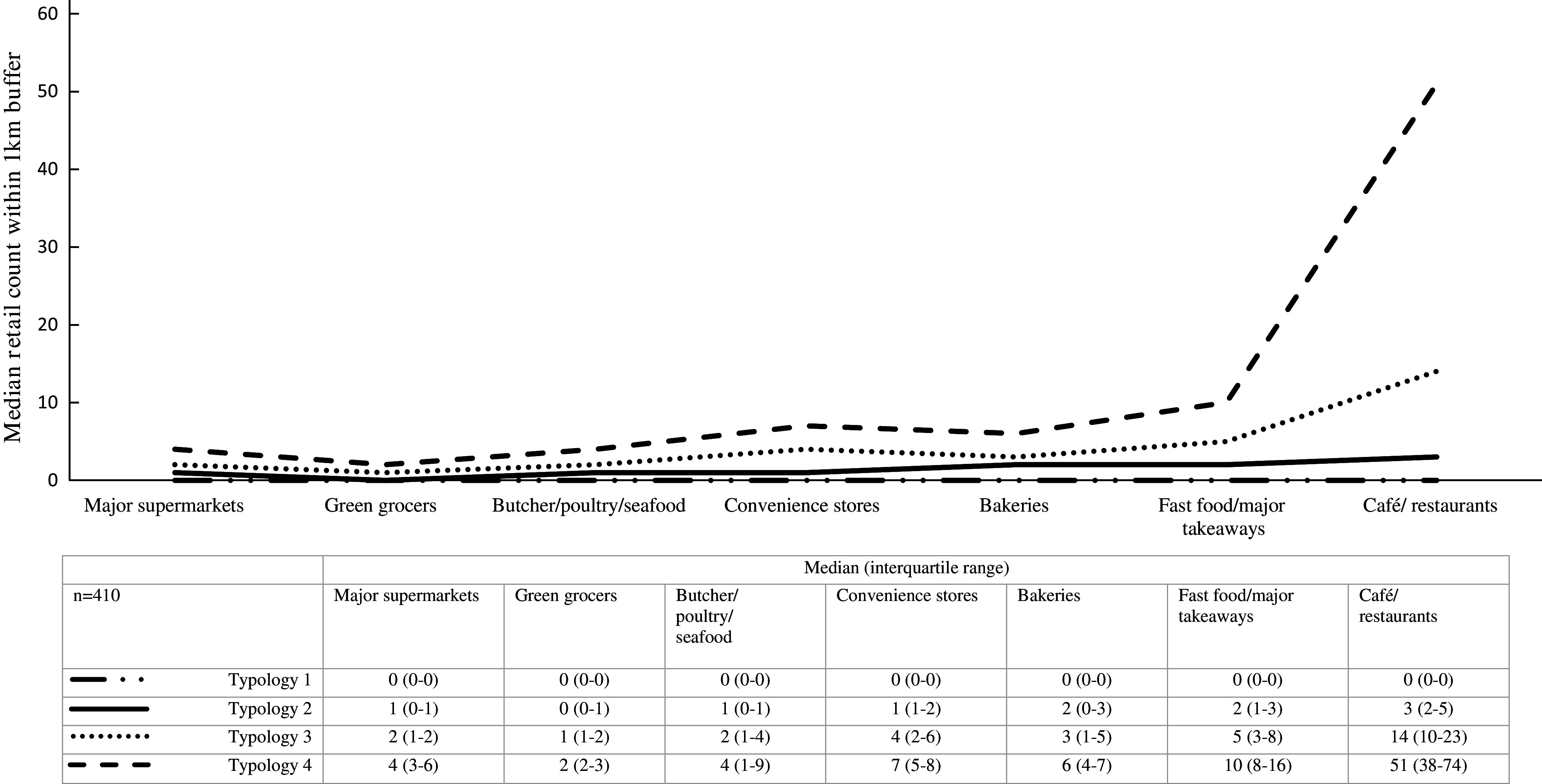
Median and interquartile range of food outlet counts within 1 km street network buffer by neighbourhood typologies (4-class solution)

Figure 1 presents the median distribution and interquartile range for each food outlet within the four typologies in the sample, and Table 2 shows the number of participants in each typology and the percentage of participants within each typology that have availability (≥ 1) of each type of food outlet. The variety and number of food outlets increases from Typology 1 to Typology 4 (Fig. 1). In particular, the largest difference was observed for café/restaurants and fast food/other major takeaways from Typology 1 to Typology 4. Typology 1, the most prevalent (52 %), is characterised by having the least number and limited variety of food outlets, but with relatively higher availability of convenience stores, café/restaurants and fast food outlets/major takeaways compared with supermarkets, bakeries and green grocers (Table 2). Typology 2 (10 %) is characterised by having some variety, with relative higher availability of café/restaurants, convenience stores and fast food outlets/major takeaways, but low median counts of food outlets; Typology 3 (32 %) is characterised by having all types of outlets present, particularly fast food/major takeaways and cafés/restaurants; and Typology 4, the least prevalent (6 %), is characterised by having a variety and abundance of all food outlets compared with the other typologies. Although Typologies 1 and 2 had low variety, the availability of convenience stores, fast food/major takeaways and café/restaurants were more common than availability of other food outlets (Table 2). Conversely, each type of food outlet was available to the majority of participants in Typologies 3 and 4. No differences were found between the four typologies and neighbourhood disadvantage (F = 0·96, *P* = 0·41).

**Table 2:** Percentage of participants (*n* 410) in each typology with availability (at least one) of each food outlet within 1 km buffer

*n* 410	Typology 1: Limited variety/low number of food outlets *n* 213 (52 %)	Typology 2: Some variety/low number of food outlets *n* 133 (32 %)	Typology 3: High variety/medium number of outlets *n* 39 (10 %)	Typology 4: High variety/high number of food outlets *n* 25 (6 %)
Supermarket	14·7	69·2	87·2	100·0
Green grocer	1·8	36·9	87·2	96·0
Butcher/poultry/seafood	6·1	51·2	77·0	84·0
Convenience stores	36·7	79·7	87·2	96·0
Bakery	8·0	72·2	89·8	96·0
Fast food/major takeaway	16·5	83·5	94·9	100·0
Café/restaurant	21·2	94·7	100·0	100·0

Associations between each of the four neighbourhood food environment typologies and fast food outlet visitation and snack food purchasing behaviour are shown in Table 3. Compared with those living in neighbourhoods with a variety and abundance of food outlets (Typology 4), there was some evidence that those living in a neighbourhood characterised as having the lowest number and variety of food outlets (Typologies 1 and 2) had three times higher odds of visiting fast food outlets once or more a week. No evidence of associations was found between neighbourhood food typologies and snack foods purchasing on the way to and from school.

**Table 3:** Odds ratios (95 % CI) of the associations between neighbourhood food typologies, fast food visitations and purchasing behaviours among adolescents (*n* 410)

				Snack food purchasing					
	Fast food visitation ≥ once/week			Bought something on the way to school ≥ once/week			Bought something on the way from school ≥ once/week		
Typologies	OR	95 % CI	*P*-value	OR	95 % CI	*P*-value	OR	95 % CI	*P*-value
Typology 4: High variety/high number of food outlets (*Reference category*)	1·0			1·0			1·0		
Typology 3: High variety/medium number of food outlets	1·78	0·51, 6·23	0·36	1·09	0·24, 4·76	0·90	1·21	0·40, 3·66	0·52
Typology 2: Some variety/low number of food outlets	3·65	1·21, 10·99	0·02	0·86	0·19, 4·00	0·85	1·38	0·44, 4·29	0·57
Typology 1: Limited variety/low number of food outlets	3·71	1·23, 11·19	0·02	1·07	0·18, 6·25	0·93	1·52	0·41, 5·57	0·52

All models adjusted for age, sex, neighbourhood disadvantage and clustering within school.

## Discussion

This study used a data-driven approach to characterise neighbourhood food environments and examine associations with fast food outlet visitation and snack food purchasing behaviour among adolescents in Melbourne, Australia. Four typologies of neighbourhood food environments were identified: the least number and limited variety of food outlets (Typology 1); some variety but low numbers of food outlets (Typology 2); variety of all food outlets present, with relatively more fast food/major takeaways and cafes/restaurants (Typology 3) and a variety and abundance of all food outlets (Typology 4). We found that those living in neighbourhoods with less variety and fewer food outlets were more likely to visit fast food outlets once or more a week compared with those living in neighbourhoods with an abundance of food outlets of all types. However, no evidence of associations was found between neighbourhood food environment typologies and snack food purchasing behaviour on the way to and from school.

In this study, more than half of the sample lived in neighbourhoods characterised as having the least number and limited variety of food outlets (Typology 1). This is similar to another study conducted with children in the same city, which also found that most children in the sample lived in neighbourhoods with little variety of food outlets^(30)^. The low number and diversity of food outlets within 1 km of participant homes observed in this study may be a reflection of the relatively small 1 km buffer. It is also possible that many of these 1 km buffers represented predominantly residential land uses. Larger buffer sizes (e.g. 2 km and 3 km) and other types of geographical buffers (e.g. sausage buffer, Euclidian buffer) may have impacted the number of food outlets captured for each latent profile in the study^(41)^. For example, a recent study compared the use of Euclidian buffer (circular buffer created by drawing a line out from a given distance from home address to form a circle) *v*. sausage network buffer (line-based buffers along all street networks at a given distance from home) and found that the sausage buffer showed a more robust positive association between the count/density of businesses and minutes of walking per week^(42)^. There are policy guidelines recommending that for growth areas in the state of Victoria, where Melbourne is situated, at least 80 % of residents should have access to a supermarket within 1 km^(43)^; however, similar policy guidelines for supermarkets and other types of food outlets for other local government areas in Victoria are yet to be implemented^(13)^. In this study, conducted across Melbourne, only a small number of adolescents were living in neighbourhoods characterised as Typology 4, where all had a supermarket within 1 km, whereas only 15 % adolescents in Typology 1 (the most prevalent typology) had a supermarket within 1 km. Several jurisdictions around the world have introduced planning policies to limit certain types of food outlets ^(44,45)^. For example, some municipalities across the USA have legislated zoning bans on fast food outlets and drive-through services ^(44,45)^. Similarly in Ireland, ‘No Fry Zones’ within 400 m around schools have been implemented ^(46)^. However, policies that consider a range of food outlets appear rare.

In our study, we found that adolescents living in neighbourhoods characterised as Typologies 1 and 2 (little variety but relatively greater availability of convenience stores and fast food outlets compared with other outlets) were much more likely to visit fast food outlets once a week or more compared with those living in Typology 4, which had the widest variety and the greatest abundance of each type of outlet. Prior research indicated that food purchasing decisions are not made merely based on awareness of one type of food outlet available, but are made in consideration of other potential alternatives within the neighbourhood food environment^(14)^. It is possible that adolescents visited fast food outlets more frequently in neighbourhoods characterised as Typologies 1 and 2 because there were a lack of other options available. For example in Typology 1, fewer than 10 % had access to green grocer/butcher, bakery. This finding is similar to a study that examined the association between relative density of fast food outlets within 10-min walk of residential areas and body weight status: they found that adults living in a neighbourhood with a high proportion of fast food outlets (five outlets and above) relative to other food outlets were 2·5 times more likely to be obese^(25)^. Other possible mechanisms for this association could be due to social normalisation of fast food visitation after school with peers^(47,48)^, higher demand for fast food due to preference^(49)^, affordability of fast food or lower price due to higher competition between fast food outlets^(50)^.

Although one in five adolescents reported to have bought snack food on the way home from school once a week or more, there was no evidence of associations between neighbourhood food typologies and snack food purchasing behaviours on the way to or from school. The lack of association may be due to the small geographical scale (1 km buffer) applied to characterise the neighbourhood food environment, as the exposure measure did not account for what adolescents actually experience en-route to and from school. It is also possible that most purchasing occurs with peers within close proximity to school with may have been beyond the 1 km buffer. Previous studies that used Global Positioning Systems to track activity spaces have confirmed the importance of environmental exposure on dietary behaviours ^(15,51,52)^. Sadler *et al.* ^(15)^, for example, examined exposure to ‘junk food’ outlets during adolescents’ trips to and from school using combined data from the Global Positioning Systems and Geographic Information Systems and found that the number of minutes adolescents was exposed to junk food outlets was positively associated with junk food consumption/purchasing behaviour en-route to and from school ^(15)^.

### Study implications

Findings from this study highlight that a variety and abundance of all types of food outlets may potentially reduce the propensity to visit fast food outlets among adolescents. The local exposure to the concomitant presence of a high number of potentially ‘healthy’ and ‘unhealthy’ food outlets may affect adolescents’ food purchasing decisions, with healthy food options potentially competing with the unhealthy food options. Conversely, local exposure to environments with less variety and predominantly fast food outlets may increase adolescents’ propensity to visit fast food outlets through cumulative exposure, which may ultimately contribute to normalisation of fast food consumption. Therefore, a concurrent consideration of the optimal mix of retail food outlets in the neighbourhood environment may be more effective in promoting healthy food choices than solely targeting a single type of food outlet (e.g. fast food outlet).

### Strengths and limitations

Strengths of this study include the assessment of a wide variety of food outlets using objective data, not just a specific type of food outlet in isolation. The use of LPA may offer a more comprehensive representation of the food environment than other approaches such as relative measures (ratio or proportion) of the food environment. However, given that the LPA approach is data driven, there may be a lack of generalisability to other jurisdictions as other data will likely result in different typologies. Thus, our findings on neighbourhood food typologies may be specific to Melbourne only, and the generalisability of our study findings will likely depend on a city’s similarities to Melbourne. Also, the study was not based on an a priori protocol or registered prospectively and uses data collected in 2014 and 2015. Information on exact response rates for individual students are unavailable for the study, and this may have implications on study generalisability. The cross-sectional design of our study means that claims about causality cannot be implied. A longitudinal design or natural experiment (e.g. opening or closing of certain food outlets) would have strengthened the study findings. The reliance on self-reported fast food visitation and snack food purchasing behaviours may be subject to recall bias. Of note, these behaviours may have occurred outside the 1 km buffer from home, particularly given that, on average, journeys to secondary school in Melbourne are greater than 7 km^(53)^. The dichotomisation of the fast food visitation and snack food purchasing variables in the analyses may have over- or underestimated the extent of variation in outcome between groups ^(54)^. Further, our study only examined snack food purchasing to and from school, and purchasing of snack foods outside of school-related travel (e.g. weekends, at night or during school holidays) was not examined. We also examined fast food visitation, rather than purchasing. It is possible that some adolescents visit fast food outlets for social reasons, without making purchases. In addition, we have focused only on typologies of food environments near home. Food outlets in other environments where adolescents spend time, such as in the area around school, may also be important. For example, evidence from activity space studies suggested that food environments other than the home environment, such as school or places where adolescents socialise or are active, are important settings for adolescents’ dietary behaviours ^(15)^. Future research should consider the use of ecological momentary assessment (i.e. real-time surveys to assess participants’ ongoing experiences and interactions) ^(55)^ with geographic momentary assessment (i.e. GPS tracking, wearable camera) ^(56)^ to further unpack the complexity of food environments and behaviour interactions among adolescents over time. It is also important to acknowledge that food outlets, including supermarkets, stock a range of foods and options, some of which could be considered healthy and others unhealthy. Thus, we did not designate outlets as ‘healthy’ or ‘unhealthy’. Point of choice marketing, product placement techniques and price and promotions can impact the sales of discretionary food in supermarkets ^(57,58)^, especially when the purchasing decisions are unplanned ^(59)^. In addition, it is important for future research to explore whether food environment typologies differ by neighbourhood socio-economic disadvantage in other cities.

## Conclusion

Using a data-driven approach, our study found four distinct neighbourhood food typologies surrounding adolescents living in Melbourne. Adolescents living in neighbourhood typologies characterised by having limited variety and a low number of food outlets but relatively greater number of fast food outlets and convenience stores were more likely to visit fast food outlets than those living in neighbourhoods characterised by having both variety and an abundance of food outlets. The findings highlight important implications for local government, planners and other stakeholders involved in the regulation, modification and management of adolescents’ food environments. In particular, a collective consideration of the overall mix of retail food outlets to reduce the propensity for fast food visitation among adolescents may be important.

## Supplementary material

For supplementary material accompanying this paper visit https://doi.org/10.1017/S1368980021004298. click here to view supplementary material
